# Cigarette smoke induced autophagy-impairment regulates AMD pathogenesis mechanisms in ARPE-19 cells

**DOI:** 10.1371/journal.pone.0182420

**Published:** 2017-08-02

**Authors:** Viren Kumar Govindaraju, Manish Bodas, Neeraj Vij

**Affiliations:** 1 College of Medicine, Central Michigan University, Mt Pleasant, Michigan, United States of America; 2 Department of Pediatrics and Pulmonary Medicine, The Johns Hopkins University School of Medicine, Baltimore, Maryland, United States of America; University of Missouri-Columbia, UNITED STATES

## Abstract

Age related macular degeneration (AMD) is one of the leading causes of blindness. Genetics, environmental insult, and age-related factors all play a key role in altering proteostasis, the homeostatic process regulating protein synthesis, degradation and processing. These factors also play a role in the pathogenesis of AMD and it has been well established that cigarette smoking (CS) initiates AMD pathogenic mechanisms. The primary goal of this study is to elucidate whether CS can induce proteostasis/autophagy-impairment in retinal pigment epithelial (RPE) cells. In our preliminary analysis, it was found that cigarette smoke extract (CSE) induces accumulation of ubiquitinated proteins in the insoluble protein fraction (p < 0.01), which was subsequently mitigated through cysteamine (p < 0.01) or fisetin (p < 0.05) treatment. Further, it was verified that these CSE induced ubiquitinated proteins accumulated in the peri-nuclear spaces (p<0.05) that were cleared- off with cysteamine (p < 0.05) or fisetin (p < 0.05). Moreover, CSE-induced aggresome-formation (LC3B-GFP and Ub-RFP co-localization) and autophagy-flux impairment was significantly (p<0.01) mitigated by cysteamine (p<0.05) or fisetin (p<0.05) treatment, indicating the restoration of CSE-mediated autophagy-impairment. CSE treatment was also found to induce intracellular reactive oxygen species (ROS, p < 0.001) while impacting cell viability (p < 0.001), which was quantified using CMH_2_DCFDA-dye (ROS) and MTS (proliferation) or propodium iodide staining (cell viability) assays, respectively. Moreover, cysteamine and fisetin treatment ameliorated CS-mediated ROS production (p < 0.05) and diminished cell viability (p < 0.05). Lastly, CSE was found to induce cellular senescence (p < 0.001), which was significantly ameliorated by cysteamine (p < 0.001) or fisetin (p < 0.001). In conclusion, our study indicates that CS induced proteostasis/autophagy-impairment regulates mechanisms associated with AMD pathogenesis. Moreover, autophagy-inducing drugs such as cysteamine or fisetin can ameliorate AMD pathogenesis mechanisms that warrant further investigation in pre-clinical murine models.

## Introduction

Age related macular degeneration (AMD) is a disorder that stems from genetic and environmental causes that initiates the degeneration of retinal pigment epithelial (RPE) cells. Subsequently, this causes poor maintenance of the rods and cones in the photoreceptor layer and a decline in central vision. AMD is described as two subtypes, “dry” and “wet” or neovascular AMD. Dry AMD involves ARPE-19 atrophy with drusen accumulation while wet AMD involves abnormal vascular endothelial growth factor (VEGF) secretion and exudates in the photoreceptor layer, causing a decrease in the patient’s best visual acuity. Clinically, wet AMD results in a much greater decline in vision because abnormal neovascularization of the macula can result in irreversible damage to the photoreceptors due to a variety of age related changes in the retina [1]. It has been found through multiple studies that cigarette smoke (CS) is the foremost factor that increases the patient’s risk for AMD [2, 3]. In these studies, CS has been shown to modulate physical factors [4–6] as well as intracellular factors [7, 8], which are also changes that have been observed in the aging eye [1]. It is also important to note that AMD is a multifactorial disorder that stems from both genetic and environmental insults. CS exposure has been shown to promote ARPE-19-cellular senescence [9] and based on the changes that have been shown to occur with retinal aging, it is likely that both CS and age related factors play a role in AMD pathogenesis. It has been well documented that aging modulates cellular proteostasis [10, 11], and recent studies have shown that proteostasis/autophagy-impairment plays a key role in AMD pathogenesis [12–15]. It has also been shown that oxidative stress alters the ubiquitin-proteasome pathway (UPP) and as proof of concept, in COPD-emphysema models, cigarette smoke and aging contribute to proteostasis/autophagy imbalance because CS impairs autophagy and proteasome mediated degradation while inducing protein misfolding [10, 16–19], a concept that has been implicated in many retinal conditions [15]. These misfolded proteins then aggregate in aggresome bodies in the peri-nuclear region and eventually mediate cytotoxicity by inducing chronic inflammatory-apoptotic responses [17, 20–23]. Therefore, drawing from concepts shown in COPD-emphysema pathogenesis, we evaluated here whether CS-exposure directly modulates proteostasis/autophagy in RPE cells that may lead to initiation of AMD. We also tested the potential of known FDA-approved autophagy-inducing drugs to control CS induced oxidative stress and resulting autophagy-impairment in order to develop a novel therapeutic strategy for controlling AMD pathogenesis.

## Materials and methods

### Cell culture

ARPE-19 cells were cultured in DME/F-12 media with 10% fetal bovine serum and 1x penicillin/streptomycin that were grown overnight at 37°C with 5% (vol/vol) CO_2_. For experimental analysis, cells were plated in 6-well plates and incubated as above. Media was then replaced with freshly prepared CSE-media or untreated-media control, at the appropriate concentrations.

### Cigarette smoke extract (CSE) preparation

CSE was prepared by bubbling smoke from research grade cigarettes (3R4F; University of Kentucky) into 20 mL of serum free DME/F-12 media approximately 1 puff for 2–3 seconds every minute. The CSE was then sterile-filtered through a 0.22 μm syringe filter and standardized to an OD of 0.74 ± 0.05 at 320 nm and pH 7.4. This was considered 100%, which was diluted with DME/F-12 media to obtain required concentrations as described recently [24, 25]. The freshly prepared CSE was used within one hour for appropriate ARPE-19 cell treatments as indicated.

### Western blot analysis

ARPE-19 cells were harvested after indicated treatments and lysed using RIPA buffer containing 1x protease inhibitor cocktail (Pierce) and EDTA. The Bradford Protein Assay Kit was used to quantify the amount of protein in the total protein lysates. Soluble and insoluble protein fractions were separated by centrifugation at 13,000 rpm for 15 min at 4°C. Soluble (60 μg) and/or insoluble protein fractions (pellet, isolated from equal amount of protein 400 μg, for each sample) were separated on 10% SDS-PAGE and transferred to 0.45 μm nitrocellulose membranes (Bio-Rad) for immunoblotting. The membranes were blocked with 1% non-fat dry milk at room temperature for 1 hour on a rotary shaker, followed by overnight incubation at 4°C with mouse monoclonal ubiquitin (1:1000; SantaCruz) and rabbit polyclonal p62 (1:500; SantaCruz) as primary antibodies. The membranes were washed (3x) with a PBS-Tween buffer (0.1% tween-20 in 1x PBS) and incubated with 1:6000 goat horseradish peroxidase-conjugated anti-mouse antibody (Novus Biologicals) or 1:6000 goat horseradish peroxidase-conjugated anti-rabbit antibody (Novus Biologicals) for 1 hour at room temperature, followed by 3x washes with PBS-tween buffer as described above. The Clarity^™^ Western ECL substrate (Bio-Rad) was used for chemiluminescence detection of immunoblots using a LI-COR C-DiGit^™^ Blot Scanner. Images were captured using Image Studio Lite 5.0, and ImageJ 1.49o (NIH) software was used to quantify changes in protein expression.

### Immunocytochemistry

ARPE-19 cells were cultured in 6 well plates overnight and treated with the 10% CSE, cysteamine (250μM, Sigma), and/or fisetin (40μM, Sigma) for 24 hours. Media was discarded and cells were washed with 1X PBS twice. Subsequently, 500 μL of 4% paraformaldehyde was added to each well and plates were then incubated at room temperature for 30 minutes, which was then discarded and the cells washed with 1X PBS twice. Next, 0.5% Tritron-X-100 was added and incubated for 30 minutes followed by washes with 1X PBS twice. For blocking, 500 μL of normal 0.5% goat serum was added and plates were incubated for 30 minutes followed by washes with 1X PBS twice. For immunostaining, primary antibodies mouse monoclonal ubiquitin (1:500; SantaCruz) and rabbit polyclonal p62 (1:500; Santa Cruz) were added in in a single mixture of 0.5% goat serum and 0.5% Triton-X-100. The primary antibody mixture was added and incubated for 2 hours, followed by washes with 1X PBS twice. Secondary antibodies, donkey anti-mouse Texas Red (SantaCruz) and goat anti-rabbit IgG-CFL488 (SantaCruz) was added to a single mixture of 0.5% goat serum and 0.5% Triton-X-100 in the dark for 1 hour, which was then discarded and cells washed twice with 1X PBS. Finally, the nucleus was counterstained with Hoechst dye (10 μg/mL) for 10 seconds and cells were washed with 1X PBS once before capturing using the ZOE^™^Fluorescent Cell Imager (Bio-Rad).

### Autophagy reporter/flux assays

ARPE-19 cells were transiently co-transfected with ubiquitin-RFP and LC3-GFP plasmids using Lipofectamine^®^ 2000 (Invitrogen, 24 hours) in a 24 well plate, and treated with 10% CSE, cysteamine (250 μM, Sigma), or fisetin (40 μM, Sigma) for 12 hours. Images were captured using the ZOE^™^Fluorescent Cell Imager (Bio-Rad). For the autophagy flux assay, ARPE-19 cells were transiently transfected with the Premo^™^ Autophagy Tandem Sensor RFP-GFP-LC3B (Thermo Fisher) plasmid in a 24 well plate for 12 hours as described recently [24, 25], and then subsequently treated with 10% CSE and/or cysteamine (250 μM, Sigma), for 12 hours. Images were captured using the ZOE^™^Fluorescent Cell Imager (Bio-Rad).

### ROS assay

Commercially available dye, CMH_2_DCFDA (Invitrogen) was used to measure the production of reactive oxygen species (ROS) in ARPE-19 cells. These cells were grown overnight in a 96 well plate with exposure to room-air or CSE (10%) and treated with cysteamine (250 μM, Sigma) or fisetin (40 μM, Sigma) for 8 hours. Cells were then incubated with 10μM of CMH_2_DCFDA for 30 minutes. Changes in fluorescence were quantified using excitation (495 nm) and emission (515 nm) wavelengths recorded on SpectraMax M5^e^ (Molecular Probes) plate reader, and the SoftMax Pro 6.0 analysis software as described recently [24–26].

### Cell viability assays

ARPE-19 cells were grown on a 96 well plate overnight and then exposed to room-air or CSE (50%) with appropriate treatments of cysteamine (250μM, Sigma) or fisetin (40 μM, Sigma) for 24 hours. Cells were the incubated with 1.2 mM MTS (Life Technologies) reagent for 1 hour at 37°C and 5% CO_2_. Absorbance was then measured at 490 nm and data analyzed by SoftMax Pro 6.0 analysis software. For the assessment of cell viability using propidium iodide (PI) staining, cells were exposed to room-air or CSE (50%) and/or parallel treatments with cysteamine (250μM, Sigma) or fisetin (40 μM, Sigma) for 12 hours. The cells were harvested, washed with PBS (2x) and incubated with PI staining solution (10 μg/ml), and data was immediately acquired using the BD FACSAria flow cytometer using the FL2 (PE) channel. Subsequently, data was analyzed using the BD FACSDiva software as we previously described [24, 25].

### Senescence assay

ARPE-19 cells were exposed to room-air or CSE (10%) and treated with cysteamine (250 μM, Sigma) or fisetin (40μM, Sigma) for 8 hours. Next, cells were fixed with 10% fixation buffer for 7 minutes at room temperature. Finally, the cells were washed with 1x PBS twice and incubated with staining solution for 4 hours. SA-β-galactosidase positive (blue) cells were counted under a Nikon ECLIPSE TS100 microscope equipped with Infinity-2 camera (Lumenera Corporation) and Infinity Analyze 6.4.1 software as we recently described [24].

### Statistical analysis

The data is shown as mean ± SEM of each experimental group. The significance between data sets was determined using a two-tailed Student’s t-test and a p-value ≤ 0.05 was considered a significant change. Densitometry analysis was performed on the immunoblots using Image J software (NIH) and a change in the expression of each protein was normalized to β-actin loading control.

## Results

### CSE exposure induces accumulation of ubiquitinated proteins in ARPE-19 cells

**Fig 1:**
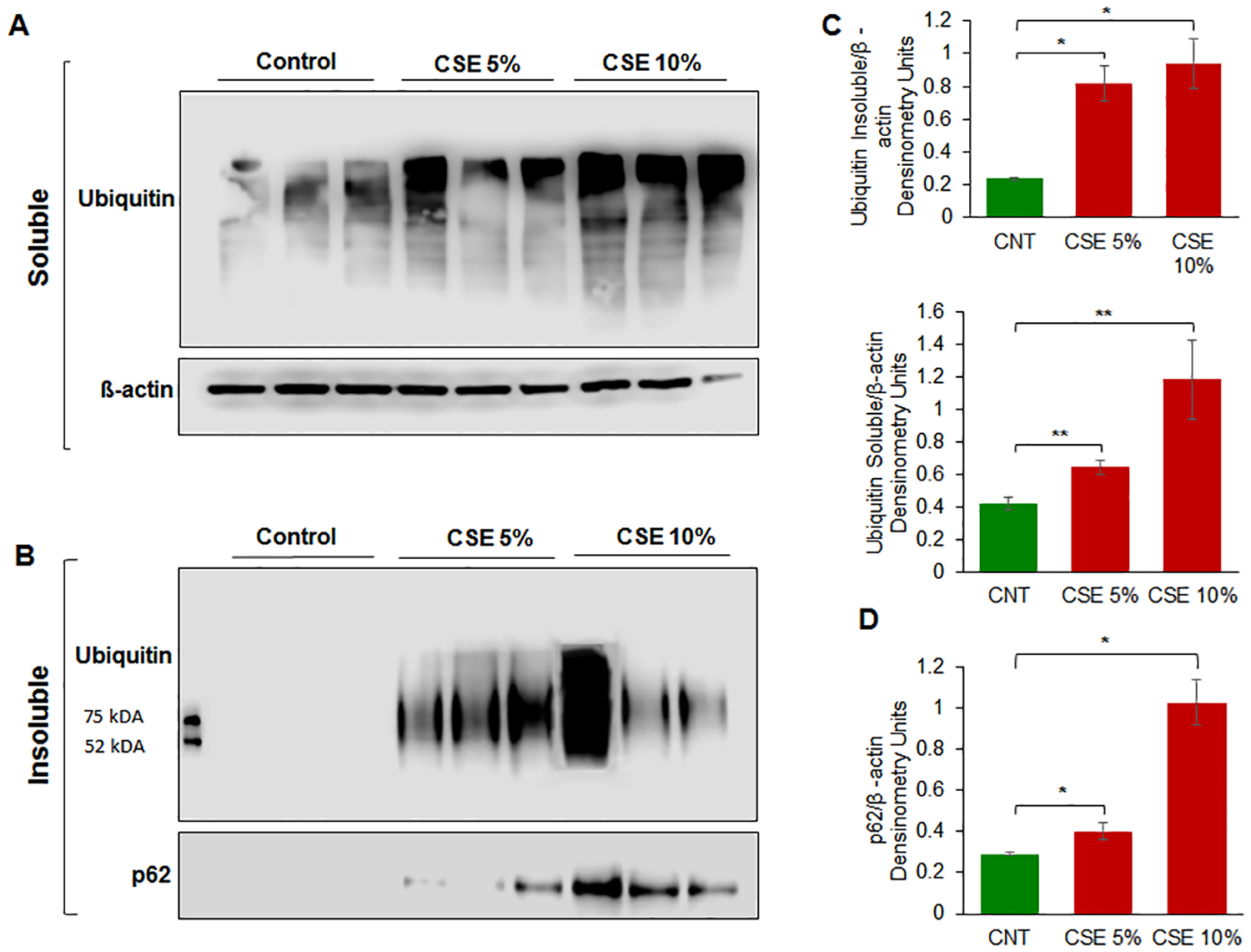
A) ARPE-19 cells were treated with the indicated doses of CSE for 12 hours. CSE was observed to increase the amount of ubiquitinated-proteins in the soluble fraction as the dose of CSE increases, indicating that CSE increases protein synthesis. B) In the insoluble fraction, we see an increase in insoluble ubiquitinated-proteins. The data suggests that CSE results in an accumulation of ubiquitinated-proteins in aggresome bodies. We also observe increase in accumulation of p62 with increasing doses of CSE, indicating that CSE induces autophagy impairment. C) Densitometry analysis shows a statistically significant (p < 0.05) increase in ubiquitinated-proteins. The insoluble fraction shows a statistically significant (p < 0.01) increase in ubiquitinated proteins, suggesting that CSE results in aggresome formation. D) Densitometry analysis of p62 shows a statistically significant increase (p < 0.01) with higher CSE doses, indicating CSE induced autophagy impairment. From the data presented as above, we can conclude that CSE induces protein misfolding, ubiquitination, and aggregation resulting in aggresome formation. Data is presented as mean ± SEM. * p < 0.05. ** p < 0.01 with n = 3 samples per group.

To identify whether CSE treatment induces autophagy-impairment in RPE cells, ARPE-19 cells were incubated with CSE (5% or 10%) for 12 hours. The protein lysates were separated into insoluble and soluble fractions and using western blot analysis, the soluble fraction was evaluated for ubiquitin (Ub) while the insoluble fraction was evaluated for both Ub and SQSTM1/p62 (p62) expression. In CSE (5% and 10%) treatment groups, we observed a distinct increase in accumulation of ubiquitinated proteins in the soluble as well as insoluble protein fractions in a dose dependent manner (Fig 1A and 1B). This suggests that CS causes proteostasis/autophagy-impairment by inhibiting or reducing degradation of ubiquitinated proteins, which results in their aggregation. In addition, we observed an increase in accumulation of p62 in the insoluble protein fraction indicative of autophagy-impairment (Fig 1B). Our data suggests that p62 cannot be cleared through autophagy as CS induces autophagy-impairment in ARPE-19 cells, similar to what has been observed for airway cells [24, 27].

### Treatment with cysteamine or fisetin mitigates ubiquitin-proteins accumulation

**Fig 2:**
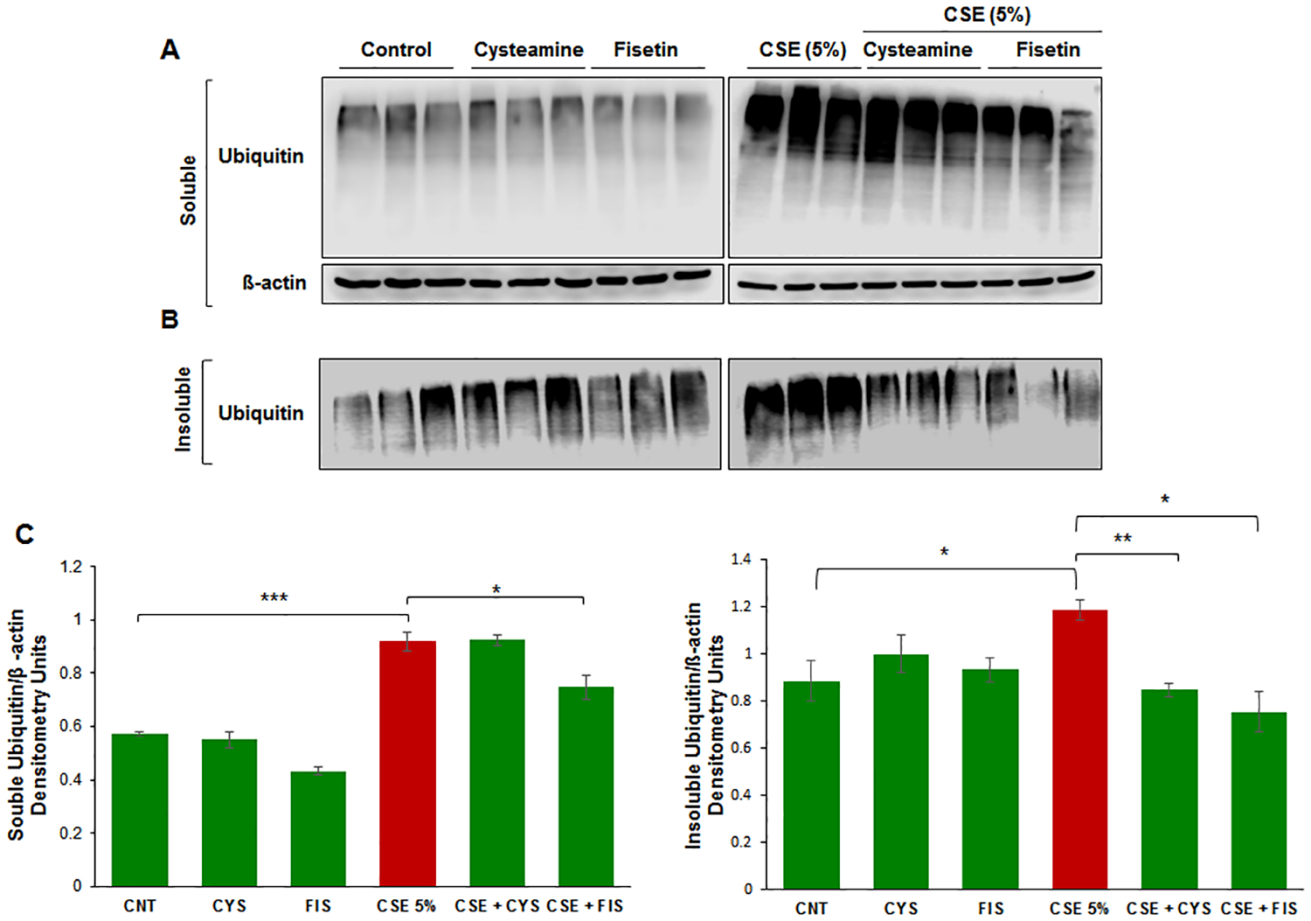
A) ARPE-19 cells were treated with the indicated dose of CSE for 14 hours. Total protein extracts were isolated and separated into soluble and insoluble fractions, which were then immunoblotted for ubiquitin. In the soluble fraction, we observed an increased accumulation of ubiquitinated proteins with CSE treatment in comparison to control. B) We observe an increase in insoluble ubiquitinated proteins when ARPE-19 cells were treated with CSE in comparison to control cells. When ARPE-19 cells are treated with cysteamine (250 μM) or fisetin (40 μM), we observed a decrease in insoluble ubiquitinated proteins. C) Densitometry analysis shows a statistically significant (p value < 0.001) increase in soluble ubiquitinated proteins with CSE treatment, and a significant decrease (p < 0.05) with only cysteamine treatment. Densitometry analysis of the insoluble fraction shows a statistically significant (p < 0.05) increase in insoluble ubiquitinated proteins with CSE treatment, and a statistically significant decrease in insoluble ubiquitinated proteins with either cysteamine (250 μM, p <0.01) or fisetin (40 μM, p < 0.05). From the data presented, we can conclude that cysteamine or fisetin treatment can ameliorate CSE induced insoluble ubiquitinated-protein accumulation in ARPE-19 cells. Data is presented as ± SEM. * p < 0.05 ** p < 0.01 *** p < 0.001 with n = 3 samples per group.

We investigated whether autophagy-induction using cysteamine or fisetin could control CSE induced insoluble ubiquitinated protein accumulation. Recent studies demonstrate the potential of both cysteamine [27] and fisetin [20] in controlling CS-induced COPD-emphysema pathogenesis *via* restoring impaired-autophagy. In the ophthalmic setting, cysteamine has been shown to downregulate inflammation [28] and fisetin has been shown to protect RPE cells from oxidative stress [29–31], which are downstream effects of CS exposure. Hence, ARPE-19 cells were incubated with CSE (5%) and/or cysteamine/fisetin for 14 hours. Cells were then lysed and evaluated for accumulation of ubiquitinated proteins in both the insoluble and soluble fractions *via* Western blot analysis. In the soluble fraction, we observed that ubiquitinated proteins accumulate under the influence of CSE that was moderately mitigated with fisetin treatment (Fig 2A). In the insoluble fraction, we observed an increase in ubiquitinated protein accumulation under the influence of CSE that was reduced by both cysteamine and fisetin treatment (Fig 2B). The CSE induced insoluble ubiquitinated protein accumulation is consistent with our results from Fig 1A. In fact, it was found that CSE induced ubiquitinated protein accumulation (p < 0.05, Fig 2C), was significantly reduced by both cysteamine (p < 0.01, Fig 2C) and fisetin (p < 0.05, Fig 2C), suggesting that cysteamine or fisetin treatment may play a role in stimulating either proteasomal degradation or autophagy in order to degrade the insoluble ubiquitinated proteins.

### CSE induces peri-nuclear localization of aggresome bodies that is mitigated by cysteamine or fisetin

**Fig 3:**
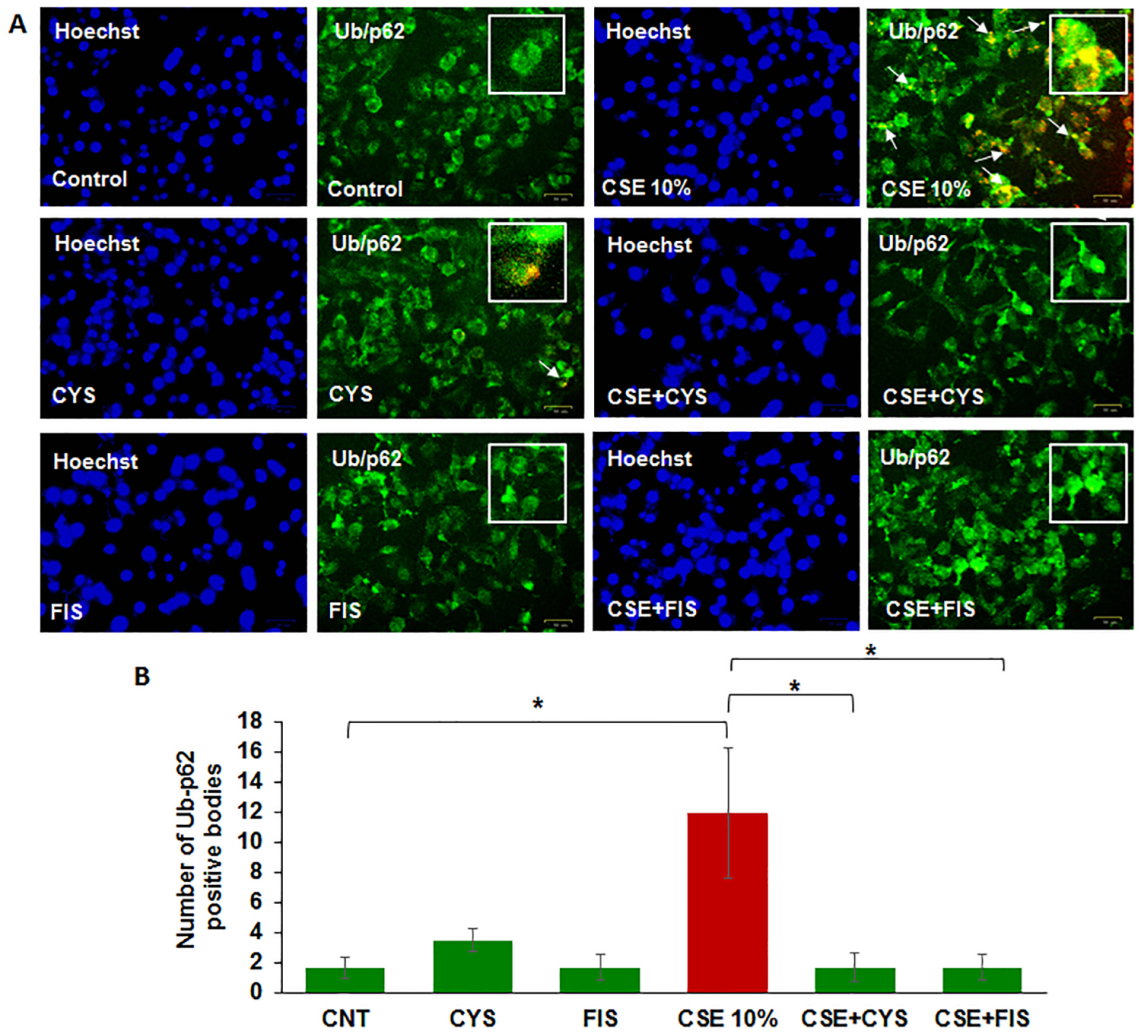
A) ARPE-19 cells were treated with cysteamine (250 μM), fisetin (40 μM), and 10% CSE as indicated in the figure for 24 hours. Immunofluorescence microscopy was done with antibodies against ubiquitin (green) and p62 (red). With CSE treatment, we observed an increase number of yellow bodies, indicating ubiquitin and p62 co-localization. Cysteamine or fisetin treatment demonstrated an observable decrease in yellow ubiquitin/p62 co-localized bodies. Comparison with the Hoechst stain showed these ubiquitin/p62 positive bodies accumulated in the peri-nuclear area. B) The number of co-localized ubiquitin and p62 bodies were counted and presented as the percentage of yellow bodies seen in the control. Analysis showed a statistically significant (p < 0.05) increase in the number of ubiquitin/p62 peri-nuclear aggregates when treated with CSE. Treatment with cysteamine (250 μM, p < 0.05) or fisetin (40 μM, p < 0.05) showed a statistically significant decrease in the number of ubiquitin/p62 peri-nuclear aggregates. From this data, we can conclude that CSE induces peri-nuclear aggregation of ubiquitinated proteins in aggresome bodies, which is ameliorated by cysteamine and fisetin treatment. Data is presented as ± SEM. * p < 0.05 with n = 3 samples per group and two images taken per sample.

Here we aim to investigate if CSE can induce the peri-nuclear aggregation of ubiquitinated proteins (aggresome bodies) in ARPE-19 cells. The cells were exposed to CSE (10%) and/or treated with cysteamine or fisetin for 24 hours, and immunostained for Ub (red) and p62 (green, impaired-autophagy marker) along with the Hoechst nuclear stain (blue). In comparison to control groups, we observed several yellow bodies in ARPE-19 cells treated with CSE (10%), indicating that there was co-localization of ubiquitinated proteins (Ub) and p62, suggestive of proteostasis/autophagy-impairment (Fig 3A) based on our previous studies in COPD-emphysema pathogenesis [17, 20, 27]. By comparing immunostained images to the Hoechst nuclear stain images, it was found that Ub-p62+ aggresome bodies accumulate in the peri-nuclear region (Fig 3A). Treatment with cysteamine or fisetin was observed to decrease the number of yellow bodies, indicating decreased Ub/p62 co-localization and peri-nuclear accumulation of these aggresome bodies (Fig 3A). Analysis showed that CSE (10%) significantly increased peri-nuclear accumulation of ubiquitinated proteins in aggresome bodies (p < 0.05, Fig 3B) that was significantly reduced by treatment with cysteamine (p < 0.05, Fig 3B) or fisetin (p < 0.05, Fig 3B). The data suggests that autophagy-inducing drugs could potentially ameliorate CS induced AMD pathogenic mechanisms.

### Cysteamine or fisetin reduce CSE induced autophagy-impairment

**Fig 4:**
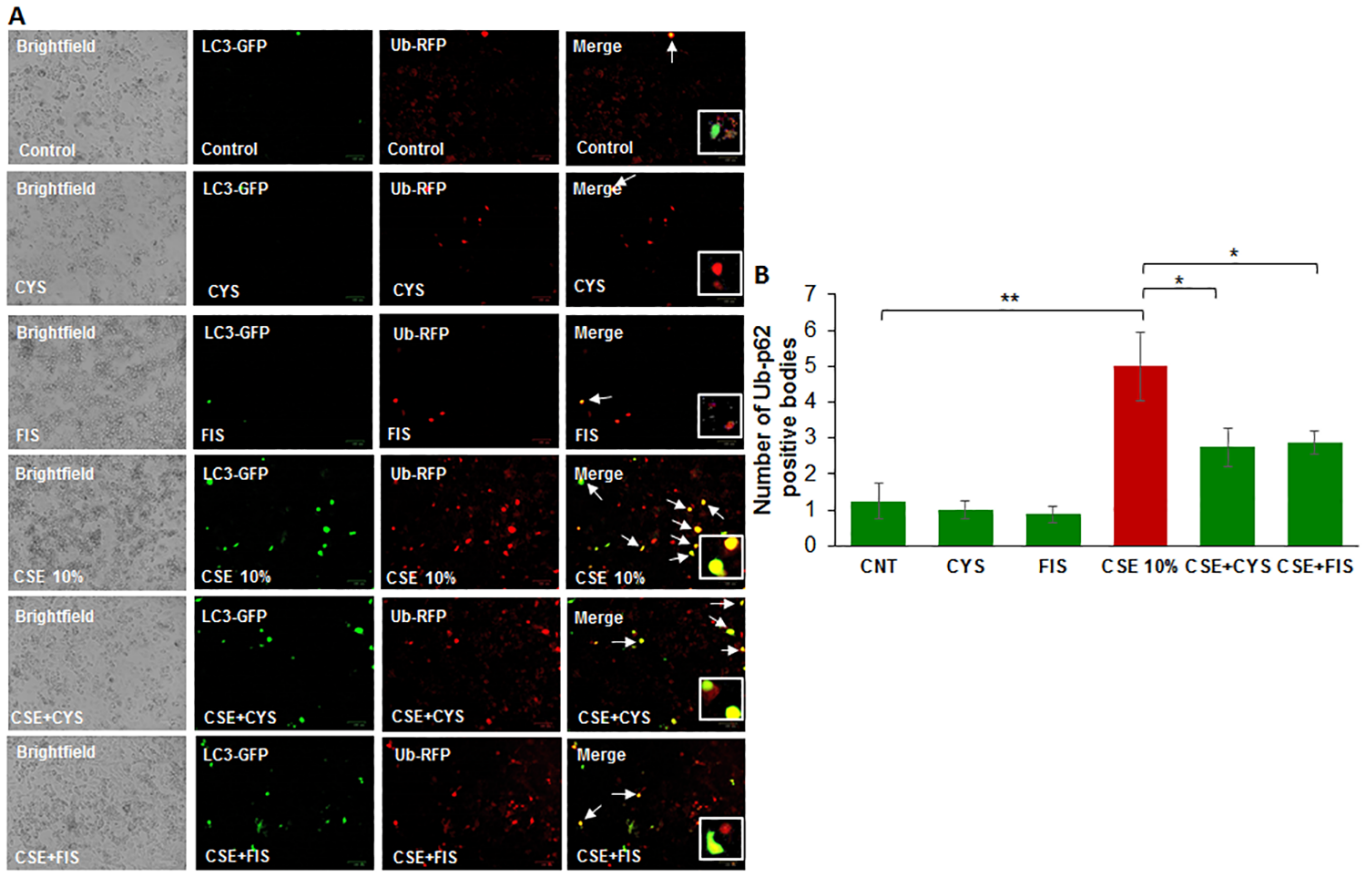
A) ARPE-19 cells were transiently co-transfected with Ub-RFP and LC3—GFP for 24 hours. Appropriate treatments of cysteamine (250 μM), fisetin (40 μM), and 10% CSE were incubated with the cells for 12 hours before image capture. CSE treatment showed an observable increase in yellow bodies, indicating LC3-GFP and Ub-RFP co-localization, which is ameliorated with cysteamine or fisetin treatment. B) The number of yellow bodies per image were counted and expressed as a percentage of control. CSE treatment induces a statistically significant (p < 0.05) increase in the number of Ub-RFP and LC3-GFP co-localized bodies, while treatment with cysteamine (250 μM) and fisetin (40 μM) both showed a significant reduction (p value < 0.05) in the number of co-localized Ub-RFP and LC3-GFP aggresome bodies. From this data, we can conclude that CSE induces aggresome body formation in ARPE-19 cells through autophagy-impairment and treatment with cysteamine and fisetin reduces the formation of aggresome bodies. Data is presented as ± SEM. * p < 0.05 ** p < 0.01 with n = 4 samples per group and two images taken per group.

To assess whether CSE treatment directly results in accumulation of ubiquitinated proteins in aggresome bodies through autophagy-impairment, ARPE-19 cells were transiently co-transfected with LC3-GFP and Ub-RFP and subsequently treated with CSE (10%) and cysteamine or fisetin for 12 hours. In comparison with control groups, the CSE (10%) treatment group showed an increase in yellow bodies, indicating LC3-GFP and Ub-RFP co-localization (Fig 4A). However, treatment with cysteamine or fisetin was observed to decrease the number of yellow bodies and therefore, an observed decrease in LC3-GFP and Ub-RFP co-localization. This data suggests that with CSE treatment, ubiquitinated proteins accumulate in the autophagosome but cannot be degraded due to autophagy-impairment. However, the decrease in LC3-GFP and Ub-RFP co-localization with cysteamine or fisetin treatment indicates ubiquitinated proteins are being appropriately cleared due to autophagy induction. Statistical analysis supports this conclusion (Fig 4B), with CSE inducing LC3-GFP and Ub-RFP co-localization (p < 0.01) while cysteamine (p < 0.05) or fisetin (p < 0.05) both reducing LC3-GFP and Ub-RFP co-localization.

### CSE-induced autophagy-flux impairment is controlled by cysteamine

**Fig 5:**
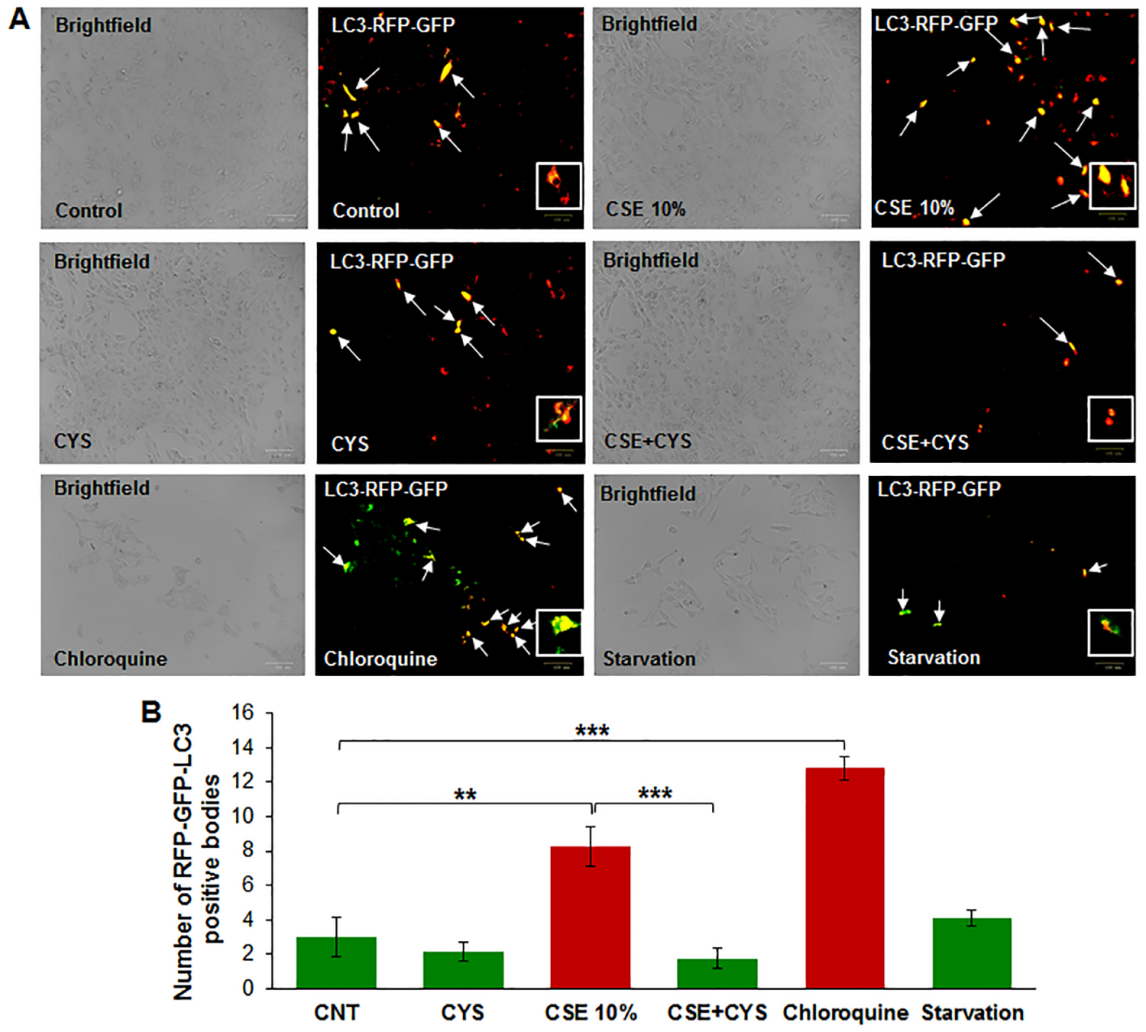
A) ARPE-19 cells were transiently transfected with RFP-GFP-LC3 plasmid for 12 hours before being treated with cysteamine (250 μM), and 10% CSE for 12 hours before image capture. CSE treatment showed an observable increase in yellow bodies, indicating lack of fusion of the autophagosome and lysosome and thus an impaired autophagy flux. Treatment with cysteamine (250 μM) showed an observable decrease in CSE-induced yellow bodies, indicating that autophagosome and lysosome fusion had taken place. B) Number of yellow bodies were counted and expressed as an average. CSE showed a statistically significant increase in yellow bodies (p < 0.01), indicating simultaneous expression of RFP and GFP and an impairment of autophagy flux. In addition, the cells were also treated with chloroquine (30 μM, autophagy-inhibitor) or subjected to serum starvation (to induce autophagy) as positive and negative controls, for which data is included in the bottom panel. Treatment with cysteamine (250 μM, p < 0.001) showed a decrease in CSE-induced yellow bodies, indicating that the acidic environment of the lysosome had inhibited GFP fluorescence, leaving only RFP fluorescence. Data is presented as ± SEM. ** p < 0.01 *** p < 0.001 with n = 3 samples for control groups and n = 4 samples for treatment groups with two images taken for each experimental group.

To evaluate CSE-induced autophagy-flux impairment, we transiently transfected ARPE-19 cells with Premo^™^ Autophagy Tandem Sensor RFP-GFP-LC3 plasmid. The presence of yellow puncta bodies indicates autophagy-flux impairment, as both RFP and GFP are simultaneously expressed due to a block in the dynamic process of autophagy (the fusion of autophagosome with lysosome to form autophagolysosome). In contrast, during the functional autophagy flux process, the GFP is degraded by the acid proteases of the autophagolysosome leaving only RFP fluorescence [32, 33]. CSE and chloroquine (inhibitor of autophagolysosome formation) treatment lead to an increase in the number of yellow bodies (Fig 5A), indicating autophagy-flux impairment. Treatment with cysteamine decreases the number of yellow bodies, indicating mitigation of CSE induced autophagy-flux impairment. Statistical analysis indicated that CSE induced autophagy-flux impairment (Fig 5B, p < 0.01) is ameliorated by cysteamine (Fig 5B, p < 0.001).

### CSE reduces ARPE-19 cell viability and induces intracellular ROS production

**Fig 6:**
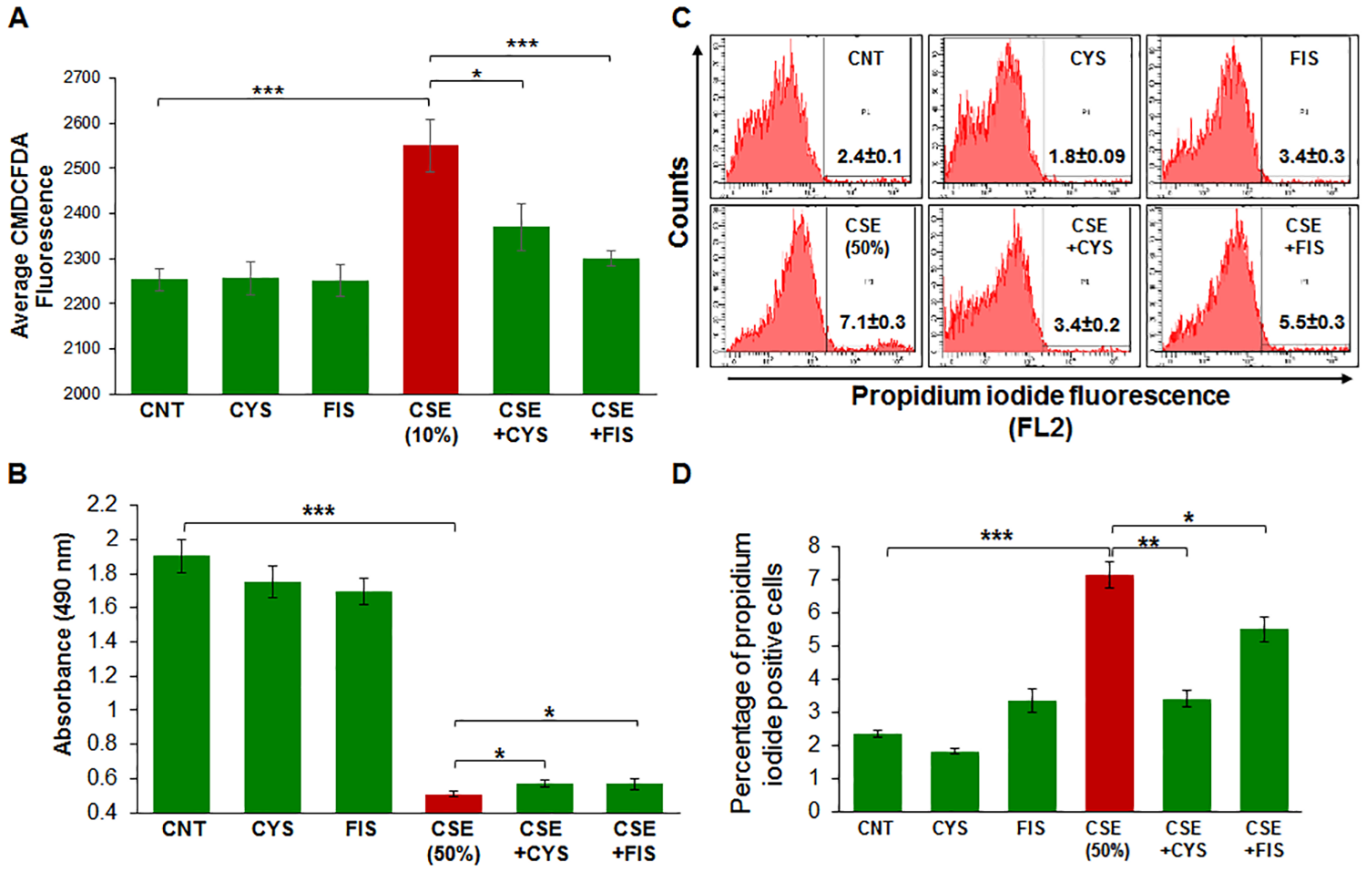
A) ROS production was assessed in ARPE-19 cells that were exposed to room-air or CSE (10%) and/or treated with cysteamine (250 μM) or fisetin (40 μM). CSE significantly induced ROS production and treatment with cysteamine (250 μM, p < 0.05) or fisetin (40 μM, p < 0.001) significantly reduced the production of ROS. The cell viability of ARPE-19 cells was assessed by MTS-based proliferation assay (B) or propidium iodide exclusion assay (C, D). Treatment of ARPE-19 cells with CSE (50%) resulted in significantly diminished cell viability (p < 0.001), while cysteamine (250 μM) and fisetin (40 μM) treatment resulted in significant rescue in cell viability and ameliorated the deleterious effects of CSE (p < 0.05). Data is presented as ± SEM. * p < 0.05 *** p < 0.001 with n = 8 samples per experimental group.

To evaluate how CSE functionally affected ARPE-19 cells, we performed a CMH_2_DCFDA assay (Fig 6A) to assess intracellular ROS production. We observed that CSE significantly increases intracellular ROS production (p < 0.001) and treatment with cysteamine (p < 0.05) or fisetin (p < 0.001) significantly mitigated intracellular ROS production. Next, we performed MTS (Fig 6B) and propidium iodide (PI, Fig 6C and 6D) staining assays to quantify changes in cell viability. First, we observed that CSE significantly diminished cell viability (p < 0.001) as observed by the MTS and PI-dye exclusion assay, while treatment with cysteamine and fisetin significantly improved the CSE-mediated decline in cell viability (p <0.05). A higher dose of CSE was used in this experiment to mimic chronic exposure of ARPE-19 cells to CSE. This data suggests that CSE functionally impairs ARPE-19 cells *via* increased intracellular ROS production and a decline in cell viability.

### CSE treatment results in ARPE-19 cell senescence

**Fig 7:**
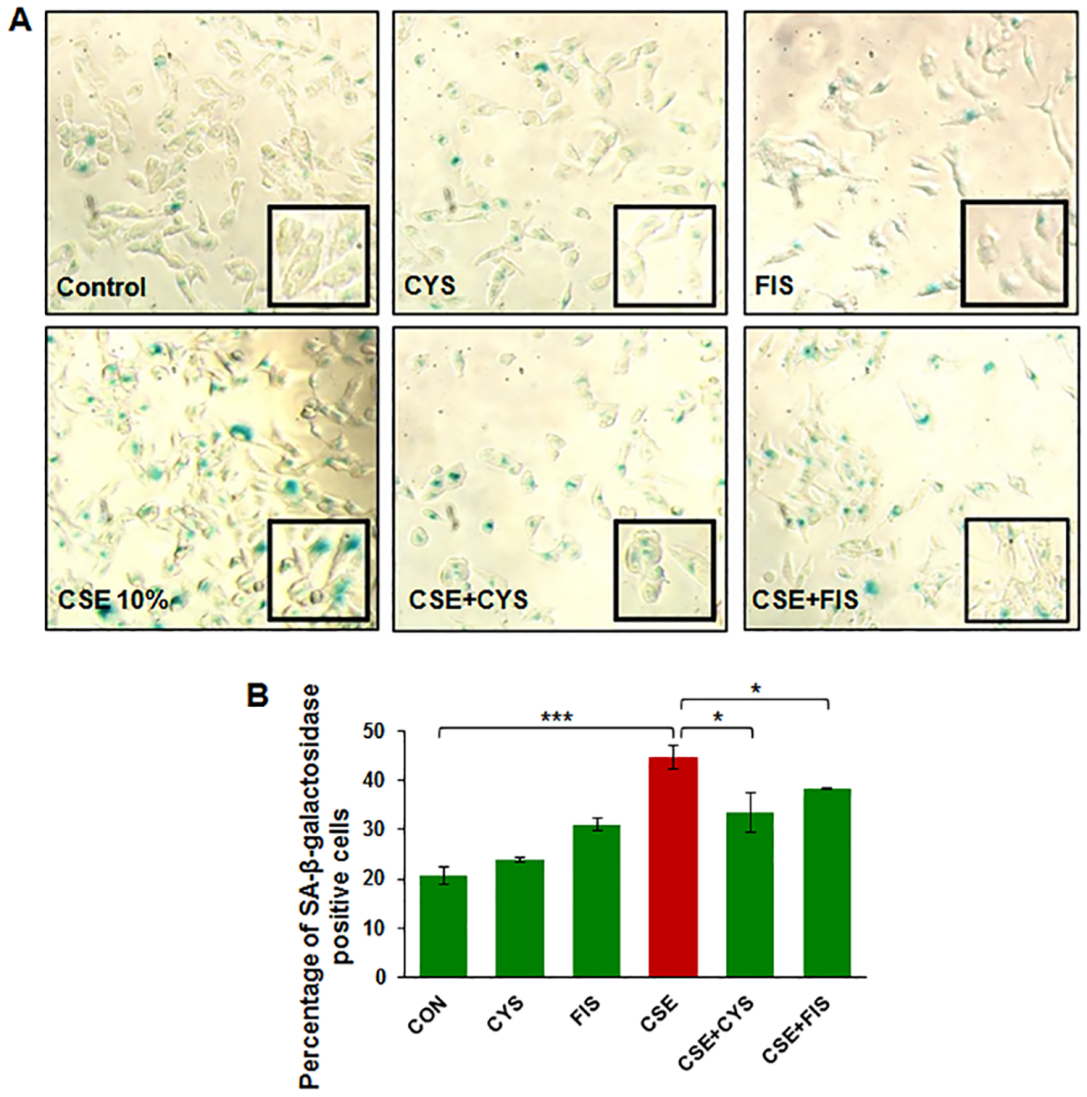
ARPE-19 cells were treated with either room-air or CSE (10%) with appropriate treatments of cysteamine (250 μM) or fisetin (40 μM). CSE (10%) was found to induce senescence, depicted by an increase in SA-β-galactosidase positive cells in comparison to the control (p < 0.001). Treatment with cysteamine (250 μM, p < 0.001) and fisetin (40 μM, p < 0.001) were found to significantly decrease the number of SA-β-galactosidase cells and therefore, reduce ARPE-19 cellular senescence. Data is presented as ± SEM. *** p < 0.001 with n = 8 samples per group.

To further assess the effects of CSE on ARPE-19 cells, we stained for senescence associated (SA)-β-galactosidase, a known marker for senescence. Cells that stained blue under light microscope were considered to be SA- β-galactosidase positive. CSE was observed to increase the number of blue cells while treatment with cysteamine or fisetin decreased the number of observed blue cells (Fig 7A). Statistical analysis showed that CSE induces senescence (p < 0.001) while cysteamine (p < 0.001) or fisetin (p < 0.001) both mitigate the number of senescent cells (Fig 7B). This data suggests that CSE induces senescence in ARPE-19 cells that can be controlled by autophagy-induction.

## Discussion

Smoking is known to be one of the leading risk factors in AMD pathogenesis [1, 15, 34], and studies have suggested that autophagy may be involved in dysfunction of RPE cells and subsequent AMD pathogenesis [11, 12, 15, 35, 36]. Moreover, in COPD-emphysema studies, smoking has been implicated in mediating autophagy-impairment and the peri-nuclear accumulation of ubiquitinated proteins as aggresome bodies that correlates with the severity of emphysema in COPD subjects [17, 20, 27]. It has also been well documented that ROS can induce ER stress and result in misfolded protein accumulation and autophagy plays a key role in alleviating ROS and ER stress accumulation [11, 37, 38]. Therefore, we hypothesized that cigarette smoking directly contributes to AMD pathogenesis by inducing proteostasis/autophagy-impairment and subsequent peri-nuclear aggregation of ubiquitinated proteins in aggresome bodies. First, we identified whether cigarette smoke exposure induces accumulation of ubiquitinated proteins and SQSTM1/p62 in the insoluble protein fractions. SQSTM1/p62 is also known as ubiquitin binding protein, and is involved in the translocation of ubiquitinated proteins to the autophagosome. Moreover, accumulation of SQSTM1/p62 in perinuclear aggresome bodies is used as an autophagy-impairment marker, indicating the autophagosome and lysosome cannot fuse [17, 20, 26, 27, 39–41]. Western blot analysis showed not only insoluble accumulation of ubiquitinated proteins but also an increase in ubiquitinated proteins in the soluble fraction in a dose dependent manner (Fig 1A), indicating that CS not only increases the ubiquitination of proteins but promotes aggregation of ubiquitinated proteins (Fig 1B). Our studies also confirmed an accumulation of SQSTM1/p62 in the insoluble protein fraction (Fig 1C and 1D), verifying autophagy-impairment that was induced by CSE. Next, we aimed to see if cysteamine or fisetin could assist in clearance of insoluble ubiquitinated proteins. Both cysteamine [27] and fisetin [20] have been evaluated in prior studies based on their known autophagy inducing properties to control CS-exposure induced COPD-emphysema pathogenesis. In our study, treatment with CS showed an increase in soluble and insoluble ubiquitinated proteins, however, treatment with cysteamine or fisetin showed a reduction in the insoluble ubiquitinated proteins (Fig 2A and 2B) on Western blot. These results suggest that if autophagy-inducing drugs can successfully clear insoluble ubiquitinated proteins, then it is likely that autophagy-impairment result in the accumulation of insoluble ubiquitinated proteins. However, we wanted to further characterize these insoluble ubiquitinated proteins and confirm that they accumulate in the peri-nuclear location similar to COPD-emphysema [17, 20, 27]. By performing immunocytochemistry for ubiquitin (Ub) and SQSTM1/p62, we verified that SQSTM1/p62 was aggregating with ubiquitinated proteins and accumulating in the peri-nuclear region. CSE was found to increase the co-localization of SQSTM1/p62 and ubiquitin, similar to our observations in COPD [24]. In comparison with our Hoechst stain (blue), these co-localized aggresomes were found accumulate in the peri-nuclear region (Fig 3A and 3B). Furthermore, treatment with cysteamine or fisetin was found to significantly reduce the SQSTM1/p62 and ubiquitin per-nuclear co-localized aggresomes (Fig 3A and 3B). These results indicate that the aggresomes are being targeted for the autophagosome but cannot be degraded and instead, accumulate in the peri-nuclear region. Prior studies have documented the increase in caspase activity [17, 20, 27] in the presence of accumulated insoluble ubiquitinated proteins in COPD-emphysema models, which is likely how peri-nuclear aggresome bodies induce cytotoxic effects on ARPE-19 cells. This evidence also further supports CS induced autophagy-impairment in ARPE-19 cells, as autophagosomes that cannot appropriately clear ubiquitinated proteins tagged with SQSTM1/p62 instead. To functionally confirm that autophagy is indeed impaired with CS exposure, we transiently co-transfected ARPE-19 cells with LC3-GFP and Ub-RFP for 24 hours and then treated them with CS. LC3 participates in stabilizing the autophagosome, and is normally degraded as the autophagosome fuses with the lysosome. Therefore, the continued visualization of LC3 indicates autophagy-impairment [41, 42]. CS exposure was found to also increase the number of LC3-GFP and Ub-RFP co-localized bodies (Fig 4A and 4B), indicating that ubiquitinated proteins are unable to be degraded through autophagy mechanisms and are continuing to remain in autophagosomes. However, treatment with cysteamine or fisetin showed a significant reduction in LC3-GFP and Ub-RFP co-localized bodies, indicating autophagy is no longer impaired. We further verified the role of autophagy-impairment in AMD pathogenesis by transiently transfected ARPE-19 cells with RFP-GFP-LC3 plasmid and analyzed for the presence of yellow bodies, indicating autophagosome and lysosome fusion had not taken place, indicating alterations in autophagy flux. Consistent with our data mentioned above, CSE had significantly impaired autophagy flux observed and quantified using number of yellow perinuclear bodies, while treatment with cysteamine significantly ameliorates CSE induced autophagy-flux impairment (Fig 5A and 5B). This evidence further verifies our hypothesis that CS exposure induces autophagy impairment in ARPE-19 cells. Our study uniquely identifies autophagy-inducing drugs as a pharmacologic treatment for improving CS induced proteostasis/autophagy-impairment in AMD, thus expanding on the current understanding of autophagy and its role in AMD pathogenesis. Next, we wished to verify how CS functionally affects ARPE-19 cells. We assessed this through CMH2DCFDA-dye based assay and MTS (or PI) assays to quantify intracellular ROS production and cell viability, respectively. CS-exposure was found to significantly increase intracellular ROS production and impair cell viability, while treatment with cysteamine or fisetin was found to significantly decrease intracellular ROS production and improve cell viability (Fig 6). Lastly, we wished to test the effects of CS on cellular senescence. Senescence of RPE cells has been identified in many studies as a key mechanism in AMD pathogenesis [43, 44], and has been shown to be induced by oxidative stress [45, 46]. In our study, we found that CS induces senescence in ARPE-19 cells, as seen by an increase in SA-β-galactosidase positive cells (Fig 7A and 7B), a result that has been studied before [9]. However, our study uniquely shows the rescue of cellular senescence with cysteamine or fisetin treatment, demonstrating the potential for these two drugs to mitigate AMD pathogenesis (Fig 7A and 7B).

## Conclusion

**Fig 8:**
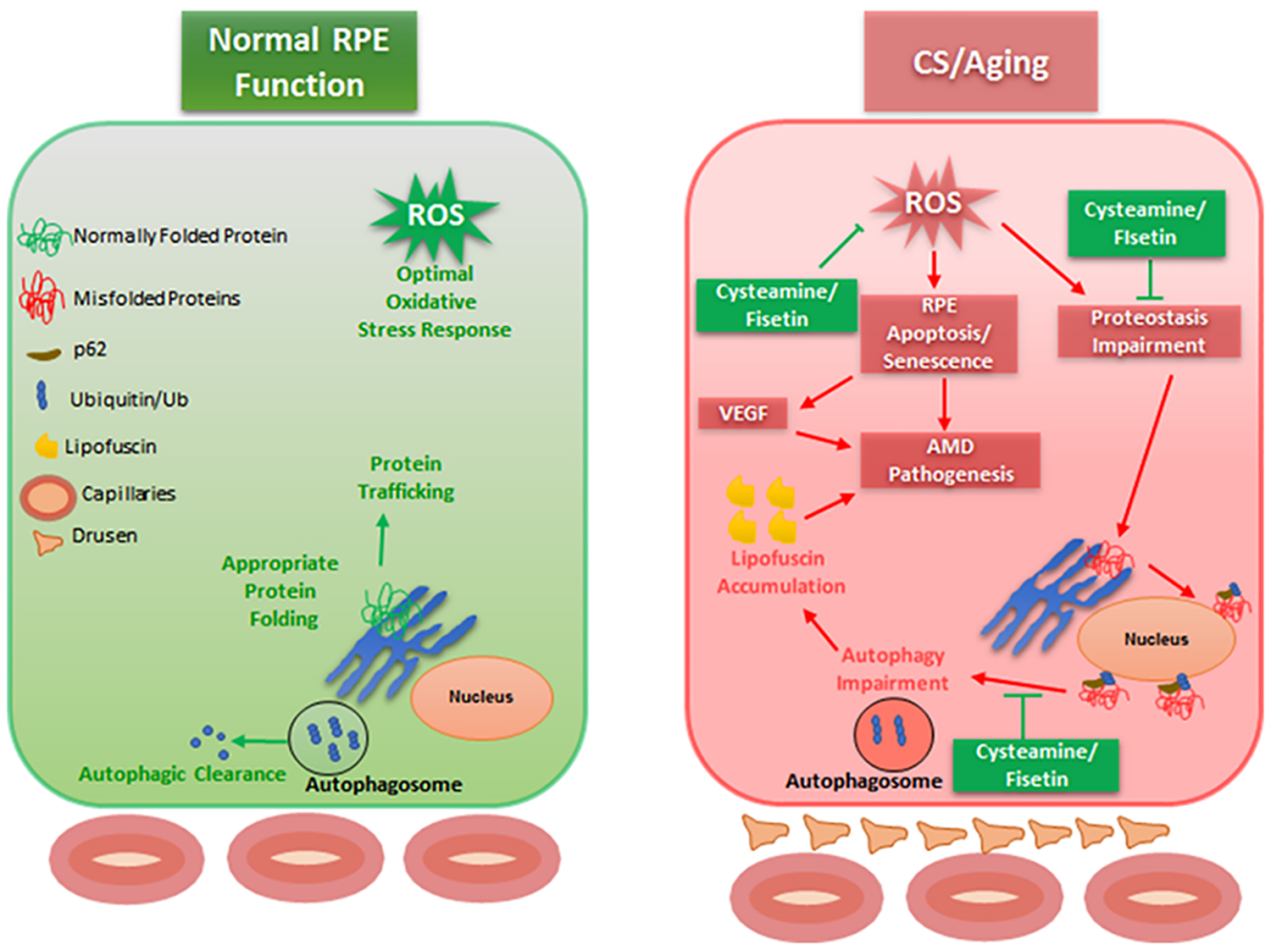
Schematic depicting proposed mechanism of AMD pathogenesis and the role cysteamine and fisetin play in augmenting proteostasis and autophagy mechanisms in CS exposed ARPE-19 cells. CS induces ROS formation in ARPE-19 cells, resulting in protein misfolding and peri-nuclear aggregation of these proteins in aggresome bodies. Moreover, these proteins cannot be appropriately degraded that eventually leads to chronic inflammatory-oxidative stress and AMD pathogenesis. Through our experiments, we have shown that cysteamine can significantly reduce ARPE-19 apoptosis while cysteamine or fisetin both can relieve ARPE-19 cells of CS induced proteostasis/autophagy-impairment.

Our study shows that cigarette smoke induces autophagy impairment in ARPE-19 cells, resulting in accumulation of ubiquitinated proteins. Prior studies in COPD-emphysema and other neurodegenerative conditions have shown that the presence of peri-nuclear ubiquitinated proteins is associated with an increase in apoptosis [20, 27, 47, 48], which is likely how ubiquitinated proteins exert their cytotoxic effects in RPE cells and contribute to AMD pathogenesis (Fig 8). Treatment of ARPE-19 cells with autophagy inducing drugs, cysteamine or fisetin reduced the CSE induced peri-nuclear accumulation of ubiquitinated proteins indicating reduction in autophagy-impairment along with controlling increased intracellular ROS production, cellular senescence and cell death, which demonstrates the potential of cysteamine and fisetin as novel therapeutic agents for AMD pathogenesis (Fig 8). Further preclinical research is warranted into the use of autophagy modulating drugs such as cysteamine and fisetin as a novel strategy for the treatment of AMD.
